# Hospitalizations for Influenza-Associated Severe Acute Respiratory Infection, Beijing, China, 2014–2016

**DOI:** 10.3201/eid2411.171410

**Published:** 2018-11

**Authors:** Yi Zhang, David J. Muscatello, Quanyi Wang, Peng Yang, Yang Pan, Da Huo, Zhongcheng Liu, Xiaojuan Zhao, Yaqing Tang, Chao Li, Abrar A. Chughtai, C. Raina MacIntyre

**Affiliations:** University of New South Wales, Sydney, New South Wales, Australia (Y. Zhang, D.J. Muscatello, A.A. Chughtai, C.R. MacIntyre);; Beijing Municipal Centre for Disease Prevention and Control, Dongcheng District, Beijing, China (Y. Zhang, Q. Wang, P. Yang, Y. Pan, D. Huo);; Beijing Research Center for Preventive Medicine, Beijing (Y. Zhang, Q. Wang, P. Yang, Y. Pan, D. Huo);; Chang Ping Centre for Disease Prevention and Control, Changping District, Beijing (Z. Liu, Y. Tang);; Huai Rou Centre for Disease Prevention and Control, Huairou District, Beijing (X. Zhao, C. Li);; Arizona State University, Phoenix, Arizona, USA (C.R. MacIntyre)

**Keywords:** Influenza, severe acute respiratory infection, hospitalization, Beijing, China, viruses, SARI, respiratory infections

## Abstract

We analyzed surveillance data for 2 sentinel hospitals to estimate the influenza-associated severe acute respiratory infection hospitalization rate in Beijing, China. The rate was 39 and 37 per 100,000 persons during the 2014–15 and 2015–16 influenza seasons, respectively. Rates were highest for children <5 years of age.

Influenza virus circulates worldwide, causing substantial rates of illness and death (*1*). In recent years, better estimates of influenza have been possible in low- and middle-income countries because of the development of surveillance systems (*2*,*3*).

Beijing is located in northern China in a temperate climate zone. Previous studies used surveillance to estimate the incidence of seasonal influenza infections in Beijing (*4*). However, hospitalizations associated with influenza have not been evaluated. We aimed to estimate the influenza-associated severe acute respiratory infection (SARI) hospitalization using the methods recommended by the World Health Organization (*5*).

## The Study

We introduced screening of inpatients for SARI at 2 sentinel hospitals in Beijing during October 2014–September 2016. Throat swabs were collected from all SARI patients with their verbal consent. To explore the characteristics of severe influenza infections, we investigated the demographic characteristics and clinical courses of SARI patients (Technical Appendix).

We estimated the rate of influenza-associated SARI hospitalizations using WHO-recommended methods (*5*). First, we defined the catchment area of the 2 hospitals. We acquired home address (village or town) of all inpatients hospitalized in 2015 from the hospital discharge registry. Villages and towns from which most (>80%) SARI patients came were defined as the catchment area. We restricted the number of SARI and hospitalized patients to patients residing in the catchment area. Second, we estimated the number of laboratory-confirmed influenza cases among SARI patients residing in the catchment area, adjusting pro rata for the proportion of SARI patients from whom specimens were obtained and tested by age group. Third, we estimated the catchment population size by 5 age groups (<5 years, 5–14 years, 15–24 years, 25–59 years, and >60 years). Catchment populations were obtained from local population statistics (*6*,*7*). Next, we obtained the age group–specific annual number of patients with physician-diagnosed pneumonia served by each of the hospitals in the catchment area by examining hospital discharge registers. We adjusted catchment population size pro rata for the proportion of pneumonia patients served by the sentinel site. The rate of influenza-associated SARI hospitalization was estimated as follows: ([number of laboratory-confirmed influenza SARI patients in catchment area ÷ proportion swabbed] ÷ [population size of catchment area × proportion of pneumonia patients served by the sentinel site]) × 100,000.

**Figure 1:**
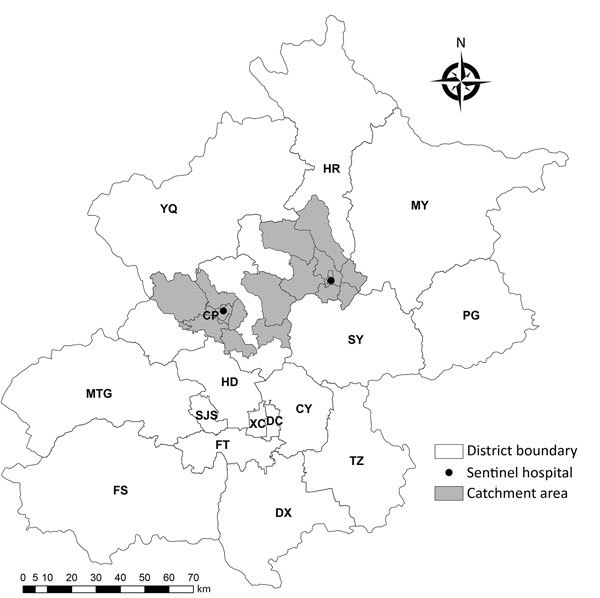
Geographic distribution of sentinel hospitals and catchment areas for surveillance of severe acute respiratory infection, Beijing, China, 2014–2016. CP, Chang Ping; CY, Chao Yang; DC, Dong Cheng; DX, Da Xing; FS, Fang Shan; FT, Feng Tai; HD, Hai Dian; HR, Huai Rou; MTG, Men Tou Gong; MY, Mi Yun; PG, Ping Gu; SJS, Shi Jing Shan; SY, Shun Yi; TZ, Tong Zhou; XC, Xi Cheng; YQ, Yan Qing.

**Figure 2:**
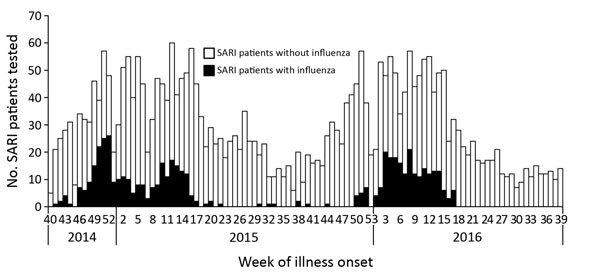
Number of total (N = 3130) and influenza-confirmed (n = 520) SARI patients from 2 sentinel hospitals combined, Beijing, China, week 40, 2014–week 39, 2016. SARI, sudden acute respiratory infection.

During the study period, 14,523 persons were hospitalized in the 2 sentinel hospitals, including 4,097 SARI patients. Eight towns were identified as catchment areas of the 2 hospitals (Figure 1,2). Of the 4,097 SARI patients, 3,899 (95.2%) resided in catchment areas and were enrolled. Swabs were collected from 3,130 (80.3%) SARI patients. Of these, 520 tested positive for influenza, resulting in a laboratory-confirmed influenza-positive proportion of 16.6%. Adjusting pro rata, the number of laboratory-confirmed influenza infections was 648 of 3,899 total SARI patients. The 2 hospitals served 93.2% of pneumonia patients in their catchment areas. Adjusting pro rata for this proportion provides a total catchment population of 842,895. Overall, the influenza-confirmed SARI hospitalization rate was 39 (95% CI 35–44) per 100,000 population during the 2014–15 influenza season and 37 (95% CI 33–41) per 100,000 population during the 2015–16 influenza season. The influenza A–confirmed SARI hospitalization rate was 24 (95% CI 21–28) per 100,000 population during the 2014–15 influenza season and 21 (95% CI 18–24) per 100,000 population during the 2015–16 influenza season; the influenza B–confirmed SARI hospitalization rate was 15 (95% CI 13–18) per 100,000 population during the 2014–15 influenza season and 16 (95% CI 14–19) per 100,000 population during the 2015–16 influenza season. In both seasons, the rate of influenza-associated SARI was highest for children <5 years of age: 335 (95% CI 277–401) hospitalizations per 100,000 population in the 2014–15 season and 529 (95% CI 456–611) hospitalizations per 100,000 population in the 2015–16 season. The rate was lowest in the 25–59-year age group: 2 (95% CI 1–6) hospitalizations per 100,000 population in the 2014–15 season and <1 hospitalization per 100,000 population in the 2015–16 season (Tables 1, 2; Technical Appendix).

**Table 1:** Rates of influenza-associated severe acute respiratory infection hospitalizations, Beijing, China

Age group, y	Cases/100,000 population (95% CI).						
2014–15 influenza season				2015–16 influenza season		
Influenza A	Influenza B	All influenza		Influenza A	Influenza B	All influenza
<5	223 (176–278)	109 (77–149)	335 (277–401)		286 (233–348)	243 (194–301)	529 (456–611)
5–14	58 (41–80)	61 (43–84)	119 (94–150)		26 (15–42)	72 (53–97)	98 (75–126)
15–24	2 (1–5)	0 (0–3)	2 (1–6)		0	0	0
25–59	5 (3–8)	4 (3–7)	10 (7–13)		3 (2–6)	1 (0–3)	4 (3–7)
>60	69 (52–88)	38 (26–53)	105 (85–129)		56 (41–74)	10 (5–20)	66 (50–86)
Overall	24 (21–28)	15 (13–18)	39 (35–44)		21 (18–24)	16 (14–19)	37 (33–41)

**Table 2:** Outcomes of SARI patients with and without laboratory-confirmed influenza. Beijing, China, week 40, 2014–week 39, 2016*

Characteristic	All SARI patients, n = 2,212	SARI patients without confirmed influenza, n = 1,759	SARI patients with confirmed influenza, n = 453	p value†
Sex				
M	1,298 (58.7)	1,030 (58.6)	268 (59.2)	0.816
F	914 (41.3)	729 (41.4)	185 (40.8)	
Age group, y				
0–4	973 (44.0)	778 (44.2)	195 (43.1)	<0.001
5–14	368 (16.6)	260 (14.8)	108 (23.8)	
15–24	40 (1.8)	35 (2.0)	5 (1.1)	
25–59	284 (12.8)	235 (13.4)	49 (10.8)	
>60	547 (24.7)	451 (25.6)	96 (21.2)	
Underlying medical condition				
>1‡	548 (24.8)	450 (25.6)	98 (21.6)	0.083
Pulmonary diseases§	224 (10.1)	175 (10.0)	49 (10.8)	0.585
Cardiovascular diseases	380 (17.2)	321 (18.3)	59 (13.0)	0.009
Metabolic diseases¶	71 (3.2)	61 (3.5)	10 (2.2)	0.175
Renal dysfunction	22 (1.0)	19 (1.1)	3 (0.7)	0.424
Hepatic dysfunction	10 (0.5)	10 (0.6)	0 (0.0)	0.108
Tumor	31 (1.4)	27 (1.5)	4 (0.9)	0.292
Immune system diseases	1 (0.1)	1 (0.1)	0 (0.0)	0.612
Received influenza vaccine within 1 y	121 (5.5)	90 (5.1)	31 (6.8)	0.15
Treatment				
Antiviral drugs	30 (1.4)	14 (0.8)	16 (3.5)	<0.001
Antibacterial drugs	2,171 (98.2)	1,722 (97.9)	449 (99.1)	0.086
Corticosteroids	214 (9.7)	154 (8.8)	60 (13.3)	0.004
Oxygen therapy	589 (26.6)	492 (28.0)	97 (21.4)	0.005
Mechanical ventilation	12 (0.5)	8 (0.5)	4 (0.9)	0.428
Complication	510 (23.1)	415 (23.6)	95 (21.0)	0.237
Pneumonia	389 (17.6)	321 (18.3)	68 (15.0)	0.106
Median length of hospital stay, d (IQR)	8.8 (8.6–9.0)	9.0 (8.7–9.3)	8.0 (7.5–8.4)	<0.001
Admission to ICU	28 (1.3)	20 (1.1)	8 (1.8)	0.286
Died	9 (0.4)	8 (0.5)	1 (0.2)	0.485

*Values are no. (%) unless otherwise indicated. The weeks range from 2014 Sep 29 through 2016 Oct 2. ICU, intensive care unit; IQR, interquartile range; SARI, severe acute respiratory infection.  †Calculated by χ^2^ test. ‡Defined as any inpatient stays with admission diagnosis in any of the following diseases, symptoms, or signs: pulmonary disease, cardiovascular disease, chronic metabolic disease, renal dysfunction, hepatic diseases, or tumor. §Asthma, chronic obstructive pulmonary disease, emphysema, chronic bronchitis. ¶Diabetes, dyslipidemia.

## Conclusions

In Beijing, influenza accounted for 16.6% of SARI in the 2 years studied; the hospitalization rate for all ages was 38–39 per 100,000 persons. This finding was much lower than that reported for Jingzhou, a city in central China, in which estimates ranged from 115 to 142 per 100,000 population in the 2010–12 influenza season (*8*). Although a similar method was used in these 2 studies, they had several differences. First, they estimated the hospitalization rate among different influenza seasons with different influenza activity and circulating strains. Second, the influenza circulation patterns differed (*9*); Beijing had 1 winter peak, whereas Jingzhou had an additional peak in summer. Moreover, the SARI definition used differed between the studies, with a lower fever threshold of >37.3°C in their study. As in other studies of age-specific influenza-associated SARI or hospitalization from other regions (*3*,*10*,*11*), we observed the most severe influenza disease in young children (<5 years). This finding underscores a need to consider influenza vaccination programs directed toward young children.

Although influenza B is often considered less severe than influenza A (*12*), 40.7% of influenza-confirmed SARI patients were influenza B–positive in this study, suggesting influenza B makes up an important component of overall influenza severity. Among the outpatient influenza infections in Beijing, 42.4% were influenza B during our study period, similar to the proportion in SARI patients (41.5%). These results suggest that influenza B is equally as responsible for mild and severe respiratory infections as influenza A.

Our study has some limitations. First, the results have limited generalizability because estimation was based on only 2 hospitals. However, 5 of the other sentinel hospitals are in the business district and serve populations of numerous districts and non-Beijing residents, making the catchment population difficult to estimate. We excluded the remaining 3 suburban sentinel hospitals because they did not have pediatric wards enrolled in SARI surveillance or the quality of their surveillance database was uncertain. Second, 19.7% of SARI patients in our study were not swabbed. We assumed the proportion of influenza-positive among them was the same as among swabbed SARI patients. However, according to clinician descriptions, most of these patients were children <5 years of age. Because the proportion of influenza-positive patients is higher among young children, we might have underestimated overall influenza-associated SARI. Third, because older adults often have complicated illness and not typical SARI symptoms, SARI might be underestimated among the older adult population. Fourth, surveillance covers only respiratory disease–related wards, but SARI patients might be hospitalized in other wards because of influenza complications (*13*,*14*), which might have led to underestimation of influenza. Finally, patients with influenza complications requiring admission might have had a relatively long delay from symptom onset to hospitalization, leading to possible false-negative laboratory results, thereby underestimating influenza.

Overall, most SARI patients in this study had influenza A, but the percentage with influenza B was also substantial. The findings of this study expanded knowledge about the impact of severe influenza and challenge the view that influenza B is a mild infection. These findings can be used to inform local policies on influenza prevention and control.

Technical Appendix Additional methods for hospitalizations for influenza-associated severe acute respiratory infection, Beijing, China, 2014–2016.
