# Supplementary material for: Hospitalizations for Influenza-Associated Severe Acute Respiratory Infection, Beijing, China, 2014–2016
Source: Emerg Infect Dis. 2018 Nov;24(11):2098–102. doi: 10.3201/eid2411.171410 (PMC6199980; doi:10.3201/eid2411.171410)
Supplement: Technical Appendix — Additional methods for hospitalizations for influenza-associated severe acute respiratory infection, Beijing, China, 2014–2016. [file 17-1410-Techapp-s1.pdf]

Article DOI: https://doi.org/10.3201/eid2411.171410

Hospitalization for Influenza-Associated Severe Acute Respiratory Infection, Beijing, China, 2014–2016

Technical Appendix

Methods

Setting

SARI surveillance has been performed in Beijing since August 2014. Surveillance is conducted in respiratory disease–related wards. This includes pediatric, respiratory, and infectious diseases wards and intensive care units (ICU). Ten general hospitals located in 10 of the 16 districts of Beijing were involved in SARI surveillance from 2014 to 2016. Among them, 5 are located in urban areas, and 5 in suburban areas. In this study, we used data collected from 2 of the 10 sentinel hospitals. The 2 chosen sites are located in suburban areas. The 2 hospitals were selected based on 1) hospitals serving patients of all ages, 2) feasibility of the catchment population estimation, 3) quality of the surveillance database reported—average weekly number of reported SARI patients >5 and confirmed influenza in at least half of the weeks during influenza peak season, and 4) average testing rate of SARI patients was >50% over the study period. The 2 selected hospitals are the largest general hospitals in each of the selected districts in terms of size and medical capability and provide ≈93.2% of pneumonia hospitalization services in each of their catchment areas. The other 8 hospitals were excluded from analysis due to disparities in data quality during the run-in period of surveillance.

Patient Enrollment and Data Collection

Physicians investigated all inpatients from the 2 selected hospitals to complete hospital admission records. They were screened for SARI during this process. SARI was defined based on WHO recommendations (*1*), “an acute respiratory infection with 1) history of fever or measured fever of $\geq$38°C; 2) cough; 3) with onset within the last 10 days; 4) and requiring hospitalization.”

The Beijing SARI surveillance protocol obliges the patient’s principal treating physicians to obtain verbal consent from eligible SARI patients, or their guardians, to collect information about demographic characteristics and clinical course within 24 hours of recognizing the patient’s SARI status. Physicians then collect the information while they investigate the patients to complete hospital admittance records. Data include date of admittance, onset of symptoms, influenza vaccination status, treatment, complications, and hospital discharge outcome. Influenza vaccination status is self-reported by the patient, or by their guardian, and verified through vaccination registries for all ages by CDC staff. Discharge outcomes are classified as death during the hospital stay or survival. Patients are not followed up after discharge.

Specimen Collection and Testing

According to the protocol for SARI, trained staff should collect throat specimens from all SARI patients with the patient’s verbal consent. Specimens were collected within 24 hours of admission and stored at 4°C at each ward before transportation to district CDCs for laboratory testing. Specimens were tested using multiplex real-time PCR to detect influenza A(H1N1 and H3N2) and influenza B(B/Yamagata and B/Victoria lineages). RNA extraction was performed from 140 μL samples using QIAamp Viral RNA Mini Kit (QIAGEN, Copenhagen, Denmark). The yield RNA was finally eluted using 50 μL RNase-free water. The viral detection was completed by rRT-PCR using AgPath-ID One-Step RT-PCR kit (Applied Biosystems, Grand Island, NY, USA) and 7500 Fast Real-Time PCR System (Applied Biosystems) using 5 μL of

RNA according to manufacturer's instructions and the WHO's protocol (*2*). All reactions were run in duplicate. Only those samples that had a cycle threshold cutoff value of <35 or >37 in duplicated tests were regarded as positive or negative, respectively. Otherwise, a retest would be performed to get a confirmed positive or negative result.

Results

Characteristics of Influenza and Noninfluenza SARI Cases

Of the 3,130 SARI patients with swab collected, 918 (29.3%) refused to be investigated for clinical information. Among the 2,212 (70.7%) SARI cases with additional demographic and clinical information available, the majority were young children <5 years old (44.0%) or older adults aged ≥60 years (24.7%). Males accounted for 58.7% of the total SARI cases. Around one quarter (24.8%) had at least 1 underlying medical condition. and 5.5% received the seasonal influenza vaccine. The median duration of hospitalization for SARI cases was 8.8 days (interquartile range [IQR] 8.6–9.0) and 9 cases (0.4%) died during hospitalization.

We compared the clinical characteristics of SARI patients by age group. The proportion of patients with underlying medical condition increased with increasing age, with the highest proportion among older adults (≥60 years) at 83.2%. The median duration of hospitalization for SARI cases also increased with age, ranging from 6 days for <5 years children to 12 days for ≥60 years adults. The 9 deaths were all older adults with a median age of 77 years (IQR 72–80).

When comparing SARI cases with and without confirmed influenza (N = 2,212); age, certain medical conditions, treatments and length of hospital stay were significantly different between the 2 groups. SARI cases without confirmed influenza were more likely to have a cardiovascular disease (18.3% versus 13.0%), were more likely to receive oxygen therapy (28.0% versus 21.4%), and were less likely to receive antivirals (0.8% versus 3.5%) and corticosteroids (8.8% versus 13.3%), compared to SARI cases with confirmed influenza. Length

of hospital stay was also significantly higher in SARI cases without confirmed influenza ($p<0.001$). The proportion of cases admitted to ICU or who died during hospitalization were not statistically significant different between influenza cases and noninfluenza cases (Table 2).

Of the 2,212 SARI patients who were investigated for clinical information, 453 were test positive for influenza viruses. We compared the severity of influenza A to influenza B infection. Around 2.6% (7/265) of influenza A–infected patients were admitted to ICU, compared with 0.5% (1/188) influenza B SARI patients. One patient (0.4%) with influenza A infection died during hospitalization, while all influenza B–infected patients were discharged. The characteristics of SARI patients (N = 2,212) and comparison between influenza- and noninfluenza-associated SARI patients are showed in Table 2 and supplementary result 1.

Of note, we found a difference in the age distribution of investigated and noninvestigated SARI patients. A lower proportion of older adults (≥60 years) was observed among investigated patients (27.2% vs 39.7%). Moreover, there were a higher proportion of influenza-positive patients among investigated patients (20.5% vs 7.3%). The overall results may have limited generalizability.

References

1. World Health Organization. WHO global epidemiological surveillance standards for influenza. Jan 2014 [cited 2017 Aug 12]. http://www.who.int/influenza/resources/documents/WHO_Epidemiological_Influenza_Surveillance_Standards_2014.pdf?ua=1

2. WHO Global Influenza Surveillance Network. Manual for the laboratory diagnosis and virological surveillance of influenza. Geneva. 2011 [Cited 2016 Dec 18]. http://www.who.int/iris/handle/10665/44518
